# Arteriosclerosis: A Consideration of the Prolongation of Life and Efficiency after Forty

**Published:** 1914-12

**Authors:** 


**Arteriosclerosis: a consideration of the Prolongation of Life and Efficiency after Forty.** By LOUIS FAUGERES BISHOP, A.M., M.D. Pp. x, 383. London: Oxford Medical Publications. 1914. Price 10s. 6d.

"Arteriosclerosis is much more frequent than formerly." We prefer to say that the diagnosis of the disease has been far more frequent than formerly, especially in America, and that it has been founded far more on obscure clinical data than on the evidence of the pathological laboratory. Fifty years ago much was written on arterial hypertrophy, and a lengthy controversy took place between Sir John Simon and Sir George Johnson, in which Dr. Howship Dickinson afterwards took a leading part, as to the question whether the hypertrophy was physiological or pathological. It will now be generally admitted that both causes are concerned in it, and that the physiological evidence derived from the introduction of the various forms of blood-pressure gauges has been of far more importance in the establishment of the modern views enunciated in this book than the *post-mortem* study of the arteries themselves.

The causes and the natural history of the condition are dealt with in considerable detail, and some excellent drawings of the arteries, as they should be in accordance with the author's views, showing hypertrophy of the media and sclerosis with the same modified by hypertonicity, which seem almost too precise to have been drawn from actual specimens, even when prepared in a similar way. There must be many fallacies in specimens of this kind, depending on the mode of preparation, as there are also in the clinical evidences drawn from the various forms of sphygmometer. The many polygraphic tracings in the book are not so convincing as the originals are likely to have been, and the skiagraphs of heart and aorta are open to various interpretations other than the diagnosis of arteriosclerosis. Questions of diet are fully considered, and the author's belief is that most cases of cardio-vascular disease developing past middle life are instances of chronic food poisoning. The best method of detoxication is by serial doses of castor oil—three doses of one ounce at forty-eight hours' intervals. He insists strongly on a low protein diet, limiting the protein content of the food to fifty grams daily (equivalent to a single chop). He points out that the high protein diet of the American people is largely responsible for a world of mischief, the real cause of which has not been generally understood or even suspected until recently, and he emphasises the value of cheese as one of the chief articles of diet for the supply of the protein element. He enters fully into questions relating to the chemistry of the proteins, and quotes the opinions of American physicians in support of his views, which are largely in agreement with those of our uric acid specialist, Dr. Haig. The book is one which should be carefully studied, and it cannot fail to be of great utility in the dietetic and general management of those whose mode of life tends to the production of this widespread and far-reaching malady.

